# The Role of RNAs and microRNAs in Non-Invasive Prenatal Diagnosis

**DOI:** 10.3390/jcm3020440

**Published:** 2014-05-06

**Authors:** Antonio Farina

**Affiliations:** Department of Medicine and Surgery (DIMEC) Division of Prenatal Medicine, University of Bologna, Bologna 40138, Italy; E-Mail: antonio.farina@unibo.it; Tel.:+39-0516-363-110

**Keywords:** circulating mRNAs and miRNAs in maternal blood, prenatal screening, fetal aneuploidies, preeclampsia, congenital heart defects

## Abstract

In this paper, all possible clinical applications of circulating mRNA and miRNA for non-invasive prenatal diagnosis appearing in the medical literature so far are described. Data from the literature have also been reported and commented on along with some possible future applications.

## 1. Introduction

In 2003, Ng *et al.* [1] first reported that fetal RNA derived from the placenta could be detected in maternal plasma. Given the intrinsic instability of RNA it was suggested that circulating RNA could be stabilized through its protection from degradation in serum, even after prolonged exposure to nucleases, in apoptotic bodies [2]. This important discovery led to the possibility of relying upon such circulating mRNA molecules for clinical uses, allowing also a gender or genotype-independent approach for non-invasive prenatal gene expression profiling. Interesting as well, for diagnostic purposes, was the evidence of the rapid clearance after delivery of all the mRNA species of placental origin [1], ensuring that the target mRNAs belong to the ongoing pregnancy. In a very recent paper [3], it has been shown that for some placental derived mRNA species, the maternal profile does not depend solely on gene expression levels in placenta. Possible reasons for such a discrepancy could be related to the trophoblast localization of the mRNA (inner or outer layer) and to the possible active placental secretion of mRNA containing microvesicles for placental hemostaticpurposes and placental-maternal communication [4].

When RNA is extracted, a substantial pool of small RNA molecules can be observed. It is now clear that small RNA species, originally thought to be just a product of RNA degradation, are actually an integral part of the cellular gene expression process. These microRNAs (miRNAs) are short, 19–25 nucleotides, stranded non-coding RNAs [5] that regulate gene expression by binding to the 3′ untranslated region of the target mRNAs and are involved in various biological processes, including development [6], differentiation [7], and apoptosis [8]. miRNAs predominantly expressed in the placenta are probably involved in placental differentiation and in the maintenance of pregnancy. Chim *et al.* [7] demonstrated that placental miRNAs are detectable in maternal plasma as well, representing a novel class of fetal nucleic acid markers, and thus can potentially be used as markers for monitoring the pregnancy. The extracellular miRNA release includes two different forms: A protein-bound mechanism where the naked miRNAs are complexed with proteins and a microvesicle-enveloped form (including microvescicles, exosomes and apoptotic bodies) [9]. Furthermore, like mRNAs, placental miRNAs follow a rapid clearance after delivery Again, certain miRNA members of the so-called C19MC (the largest cluster of mRNAs in the human genome expressed predominantly in the placenta identified by Bentwich *et al.* [10]) are relatively more abundant in the maternal plasma than in the corresponding placenta, suggesting a selective mechanism to regulate their C19MC release [11].

## 2. Strategy for Systematic Identification of Placental-Derived RNA and miRNA Markers in Maternal Plasma

Considering that the placenta, or, in the first trimester, the chorionic villi, are the tissues responsible for the release of fetal RNA into maternal plasma and that the detectability of transcripts is directly related to their expression levels in the tissue under exam, the proposed strategy for detecting the circulating mRNAs of interest, was to systematically evaluate the gene expression or the miRNA profile in placental tissue and maternal blood by microarray analysis, followed by a real time quantitative RT-PCR aimed at detecting the most aberrant mRNA or miRNA species in maternal plasma in a new series of patients. Tsui *et al.* [8] used this method during both the first and third trimester of pregnancy for detecting mRNAs; the same method was later used by Chim *et al.* [7] for detecting miRNAs in maternal plasma.

## 3. mRNA and Fetal Aneuploidies

Despite the initial promising results, only a few attempts have been reported in medical literature regarding the use of placental mRNA in screening for Down’s syndrome. The rationale of such an approach was based on the dosage of mRNA specific to chromosome 21 that, due to the different expression in normal *versus* trisomic fetuses, would, in the latter, yield a higher value of mRNA expression than expected. However, for the first mRNA tested, the C21orf105 mRNA, no significant aberrant values were detected in the maternal plasma of women carrying a fetus with trisomy 21 despite its location in the Down’s syndrome critical region [12]. The lack of a significant differential data distribution was attributed to the wide biological variation between and within individuals, along with a low expression profile in maternal blood.

By using a different approach, the RNA allelic ratio assessment using SNP (RNA-SNP ratio) in a case-control study, Lo *et al.* found a different RNA-SNP ratio distribution in cases *versus* controls associated with a retrospective detection rate of 90% with a false positive rate of 4.5% [13]. The placental target gene used to calculate the deviance from the expected RNA-SNP ratio was *PLAC4*. Briefly, in the RNA-SNP ratio approach, an euploid fetus heterozygous for a certain SNP on the chromosome of interest would have an expected RNA-SNP ratio 1:1. If the fetus has a condition of trisomy 21 and is heterozygous for a SNP on a chromosome 21 transcribed mRNA like *PLAC4*, then the RNA-SNP allelic ratio would become 1:2 or 2:1.

However, a major disadvantage is that the method is polymorphism-dependent. For example, *PLAC4 S*NP has a heterozygosity rate of 0.45 in the Chinese and Caucasian population, resulting useful in providing information in only about 50% of the screened subjects. A prospective use of SNP encoded by the *PLAC4* gene was described by Tsui *et al.* [14]. In order to improve the detection rate of the target gene for heterozygous fetuses, a combined strategy involving detection of RNA-SNP by mass spectrometric (MS) and digital PCR methods was used. Instead, for pregnancies in which the fetuses were homozygous for the SNP, total *PLAC4* mRNA concentration in maternal plasma obtained by real-time PCR and digital PCR was used. Sixteen Down’s syndrome cases were matched with 137 euploid fetuses at 12 weeks gestation. Only 4 (33%) fetuses were affected by Down’s syndrome and 58 (42.3%) euploids were in the heterozygous group. For this small sample for which the RNA-SNP approach was used, the authors concluded that the detection rate was 100% with a false positive rate of 10.3% for both the MS and the digital PCR methods. Instead, for the homozygous fetuses in which real-time PCR and digital PCR quantifications were used, the quoted detection rate was only about 10%–15% with a false positive rate of 5%–10% (by visual inspection of the ROC curve). This poor result was also confirmed by Banzola *et al.* in 2008 [15].

Yang *et al.* by five SNPs on gene *PLAC4* were able to detect all the four heterozygous cases of Down’s syndrome cases out of 17 (23%) by using the multiplex SNaPshot assay method [16].

A possible new source of circulating mRNAs expressed differently in aneuploid pregnancies has been described in a paper by Rozovskiè [17]. In fact, in a genome-wide expression analysis of cultured chorionic villi in trisomy 21 affected fetuses, abnormal gene expression was only partially explained by over-expression of genes from chromosome 21 since other genes mapping on other chromosomes have been found to be aberrant. Therefore it can be speculated that other mRNAspecies not belonging to chromosome 21 could have a discriminant ability for detecting affected fetuses.

## 4. mRNA and Preeclampsia

Preclampsia (PE) is a pregnancy related disorder characterized by hypertension and proteinuria after 20 weeks of gestation. It is a leading cause of maternal and fetal mortality and morbidity worldwide. To date, delivery is the only cure for the disease.

Strategies for detecting candidate genes for the screening of the disease in the first trimester include microarray analyses in both placentas of affected cases collected after delivery and in trophoblast tissue of patients who developed PE later in pregnancy [18,19]. However, despite the many papers that report the placental gene expression profiles of PE affected patients [20,21,22,23,24,25], the vast majority of these studies are mainly addressed at explaining the pathophysiology and the evolution of the disease rather than toward the detection of genes increased in maternal plasma during the pregnancy. Also, the mRNA expression variations associated with PE-specific genes in circulating maternal blood cells has been evaluated [26].

Several papers [27,28,29,30,31,32,33,34,35,36,37,38,39] report an association between circulating mRNA species and preeclampsia (PE). The first evidence of the presence of abnormal circulating mRNA in women affected by PE was reported in a paper by Ng *et al.* [27] where, in pregnancies at term, higher levels than expected of mRNA for the *CRH* gene were found for a group of controls [27]. A second observation by Farina *et al.* [28] showed that the levels of mRNA for the same *CRH* gene, when expressed as multiples of the median (MoM), were directly related to the gestational age at the time of the blood draw and to the severity of the disease [28]. These results were also confirmed by Freeman *et al.* [29] and Galbiati *et al.* [30] and also described the correlation between the *CRH* gene and the levels of procoagulant factors such as factor VII. After these preliminary but fundamental results, many other papers, in which maternal whole blood was used, showed that many mRNA species were expressed differently in PE patients. Table 1 reports the list of the mRNA-species found to be distributed differently in PE cases *vs.* controls and their degree of aberration in the sample of studied patients [31,32,33,34,35,36,37,38,39,40,41,42]. Unfortunately, the data are collected with much variability in the population and there is no agreement regarding the week for sample collection and the stratification with the degree of severity. For some papers [32,35,36,37] HELLP cases have been included as well. However, *CRH* [27,28,29,30,33,36], *SELP* [31,36] and *PLAC1* [36,38,39] are the most studied markers with a weighted mean (SEM) increase of 6.28 (0.25), 7.84 (0.29) and 7.49 (0.75) folds respectively.

## 5. mRNA and PE Screening

Given the mortality and morbidity associated with PE, there is a need for the identification of markers that would allow early detection of patients at risk. Several biomarkers, including some molecular species that may help in identifying those women who are likely to develop PE have been proposed, alone or together. Very few studies investigate circulating fetal RNA status prior to the development of PE symptoms. The panel of examined genes included some novel species, such as *PLAC1*, *PLAC3*, *PLAC4*, *ERVWE1* and *PTX3*, but unfortunately for all of the genes studied (like *ENG*, *sFlt-1*, *PlGF*, *PP13*, *CRH*), the association between the final protein product and PE was already known. Some of them, like *PlGF* and *PAPP-A*, are in fact currently used to screen for PE in the first trimester. Only a few papers reported the detection rates of various panels of mRNA species that, in multivariable models that also included the parity, yielded a detection rate ranging from 60% to 80% at 5% FPR [40,41,42] as showed in Table 2. Currently, there is not yet a large scale trial to evaluate the ability of the most over or under expressed markers to discriminate in the screening for PE. However, it seems that *s-Flt1* and *ENG* are those with the highest detection rate.

A single observation by Anton *et al.* [43] reported the possible discriminant ability of miR-210 long before the clinical onset of the disease.

**Table 1:** mRNA species dosed in maternal plasma or whole blood variably distributed in preclampsia (PE) patients.

Gene Symbol	First Author	Population *N* Cases/*N* Controls	Mean or Median Gestational Age at Blood Test in Cases/Controls (Weeks)	Expression in PE Patients	Stratification for PE Severity
*CAT*	Nakamura, M. [35]	24/24	39/38	2 folds lower	Yes, HELLP cases included
*CRH*	Ng, E. K. [27]	12/10	37/38	10 folds higher	No
	Farina, A. [28]	17/17	36/37	9 folds higher (MoM)	Yes
	Freeman, D. J. [29]	32/32	36/36	4–10 folds higher	No
	Galbiati, S. [30]	10/12	24–36/24–36	3.5 folds higher *	No
	Paiva, P. [33]	15/15	31.1/30.1	12 folds higher	Yes (only early PE)
	Purwosunu, Y. [36]	43/41	39/39	3.94 folds higher	Yes, HELLP cases included
*ERVWE1*	Paiva, P. [33]	15/15	31.1/30.1	2 folds higher *	Yes (only early PE)
*GCM1*	Fujito, N. [39]	10/26	35.7/36.1	2.43 folds higher	No
*GPx*	Nakamura, M. [35]	24/24	39/38	4.57 folds lower	Yes, HELLP cases included
*HO-1*	Nakamura, M. [35]	24/24	39/38	5.7 folds lower	Yes, HELLP cases included
*HO-2*	Nakamura, M. [35]	24/24	39/38	17 folds lower	Yes, HELLP cases included
*hPL*	Farina, A. [31]	6/30	33/32	1.51 folds lower	No
*Inhibin A*	Farina, A. [31]	6/30	33/32	1.41 folds higher	No
*KiSS1*	Farina, A. [31]	6/30	33/32	2.11 folds higher	No
*PAI-1*	Farina, A. [31]	6/30	33/32	1.36 folds lower	No
	Purwosunu, Y. [37]	43/41	39/39	2.48 folds higher (MoM)	Yes, HELLP cases included
*PAPP-A*	Kodama, M. [38]	11/22	29/30	71 folds higher (MoM)	Yes, early onset (<34 weeks)
	Kodama, M. [38]	21/42	37.8/36.2	7 folds higher (MoM)	Yes, late onset (>34 weeks)
*PLAC1*	Purwosunu, Y. [36]	43/41	39/39	3.95 folds higher	Yes, HELLP cases included
	Kodama, M. [38]	11/22	29/30	25 folds higher (MoM)	Yes, early onset (<34 weeks)
	Kodama, M. [38]	21/42	37.8/36.2	5 folds higher (MoM)	Yes, late onset (>34 weeks)
	Fujito, N. [39]	10/26	35.7/36.1	8.69 folds higher	No
*PLAC3*	Paiva, P. [33]	15/15	31.1/30.1	11 folds higher	Yes (only early PE)
*PLAC4*	Paiva, P. [33]	15/15	31.1/30.1	4 folds higher *	Yes (only early PE)
*PP13*	Shimuzu, H. [34]	24/22	39/38.5	3 folds lower	No
*PSG1*	Okazaki, S. [32]	28/29	39/38	32.59 folds higher	Yes, HELLP cases included
*SELP*	Farina, A. [31]	6/30	33/32	2.43 folds higher	No
	Purwosunu, Y. [36]	43/41	39/39	8.60 folds higher	Yes, HELLP cases included
*PTX3*	Galbiati, S. [30]	10/12	24–36/24–36	2.50 folds higher *	No
*SOD*	Nakamura, M. [35]	24/24	39/38	3.2 folds lower	Yes, HELLP cases included
*t(PA)*	Purwosunu, Y. [37]	43/41	39/39	3.33 folds higher (MoM)	Yes, HELLP cases included
*TPBG*	Okazaki, S. [32]	28/29	39/38	11.57 folds higher	Yes, HELLP cases included
*VEGF*	Farina, A. [31]	6/30	33/32	1.72 folds higher	No

* By visual inspection.

**Table 2:** mRNA species dosed in maternal plasma or whole blood variably distributed in patients who developed PE later in pregnancy.

Gene Symbol	First Author	Population	Mean or Median Gestational age at Blood Test in Controls and in Cases (Weeks)	Detection Rate at 5% FPR	Weighted Mean Detection Rate at 5% FPR
*S-Flt1*	Sekizawa, A. [40]	62/310	17.3/17.3	43.6	-
	Purwosunu, Y. [41]	62/310	17.3/17.3	58.0	50.36
	Farina, A. [42]	11/88	12/12	45.5	-
*ENG*	Sekizawa, A. [40]	62/310	17.3/17.3	47.3	-
	Purwosunu, Y. [41]	62/310	17.3/17.3	43.5	44.67
	Farina, A. [42]	11/88	12/12	36.4	-
*t-(PA)*	Purwosunu, Y. [41]	62/310	17.3/17.3	33.9	-
*PAI-1*	Purwosunu, Y. [41]	62/310	17.3/17.3	29.0	-
*VEGF*	Purwosunu, Y. [41]	62/310	17.3/17.3	29.0	-
*tgfb1*	Farina, A. [42]	11/88	12/12	27.3	-
*PlGF*	Sekizawa, A. [40]	62/310	17.3/17.3	24.2	-
*SEL*	Sekizawa, A. [40]	62/310	17.3/17.3	18.2	21.2
	Purwosunu, Y. [41]	62/310	17.3/17.3	24.2	-
*PLAC1*	Sekizawa, A. [40]	62/310	17.3/17.3	20.0	18.9
	Purwosunu, Y. [41]	62/310	17.3/17.3	17.7	-
*HO-1*	Sekizawa, A. [40]	62/310	17.3/17.3	8.10	-

## 6. mRNA and Intrauterine Growth Restriction (IUGR)

IUGR is usually defined as a birth weight below the 10th percentile for gestational age and gender and is associated with severe perinatal morbidity and mortality. Infants with IUGR that survive the neonatal period are also at risk of neurodevelopmental problems in childhood, including cerebral palsy, as well as adult metabolic and cardiovascular disease.

Only a few papers reported the possible use of mRNA species in IUGR. Pang *et al.* [44] showed that there is a correlation between circulating mRNA for *GH2* and fetal growth in a non-stratified series of IUGR and normal fetuses.

In early euploid IUGR (20–24 weeks gestation) higher than expected values of mRNA for the *EGFL7* gene have been found by Zanello *et al.* [45]. mRNA for the *p21* and *hif1α* genes has been found to be more prevalent in pregnancies complicated by hypoxia and/or IUGR in a paper by Ashur-Fabian *et al.* [46]. Similar results were also found in a paper by Mouillet *et al.* [47], where a panel of hypoxia-regulated miRNA was found to be higher in maternal plasma of women bearing a IUGR fetus.

The most extensive results come from a paper by Whitehead *et al.* [48] where a correlation between mRNA found in maternal blood encoded by growth genes (*IGF1*, *IGF2*), *IGF1R*, *IGFBP2*, *GH2*, *ADAM12* and the severity of disease, in severe preterm IUGR, has been demonstrated. Again, some circulating mRNA species (*IGF2*, *GH2* and *IGFBP2*) were abnormal at 28–36 weeks, long before the clinical evidence of IUGR. Reduced placental expression of some miRNAs located at C19MC has been found in IUGR cases but, unfortunately, no statistical differences have been detected for the most aberrant placental tissue miRNAs when dosed in maternal plasma in a small series of 10 IUGR cases matched with 10 proper controls [49].

## 7. Other Clinical Conditions

Some circulating mRNA species including *SAV1*, *TNXB2* and *PAPP-A* have been found to be abnormal in a group of pregnancies in which the women were bearing a fetus affected by a congenital heart defect (CHD) in the second trimester of pregnancy [50]. Further support for this hypothesis comes from a subsequent observation by Yu *et al.* [51]. Again, a recent paper by Zhu *et al.* [52], showed that the expression of some maternal serum miRNA species is up-regulated in pregnant women with fetuses with CHD at 18–22 weeks gestation.

An observation by Farina *et al.* [53] reported that lower levels than those expected of *PLAC1* mRNA can be found in whole blood of women who experienced a threatened miscarriage but with a viable embryo in the late first trimester. However, if the *PLAC1* gene is related to placental development [54], its concentration in whole blood could also be derived from tissues or organs other than the placenta [55].

Finally, in placenta accrete, higher values of cell-free *CSH1* mRNA have been found [56]. More recently, mRNA for *PLAC1*, *VEGF* and *s-Flt1* genes was found to be higher, and mRNA for the *KiSS1* gene lower than expected in a single case report when compared to a group of controls [57].

## 8. Conclusions

Circulating mRNAs and miRNAs are an important source of genetic material that can potentially be used to screen and monitor some conditions specific to pregnancy. However this is an approach that still carries uncertainties. In fact, massive parallel sequencing (MPS) seems to be the best method for screening aneuploides when compared to the RNA-SNP ratio since it is a gender and polymorphism independent method and it also allows to test for many aneuploides simultaneously. So far, PE has been widely investigated by mRNA analysis, along with IUGR, but still few studies have attempted to use circulating nucleic acids as screening tools for both these diseases. Results seem encouraging, but a considerable number of species must be dosed simultaneously in order to be considered at a similar level for PE screening as the current available methods based on biochemical, Doppler, anamnestic and biophysical factors.

Finally, an interesting field seems to be the use of circulating mRNA and miRNA in some fetal malformations like CHD as a screening tool to identify those pregnancies to be addressed to fetal echocardiography.

## List of Genes and Abbreviations

**Name****Official Symbol***catalase**CAT**chromosome 21 open reading frame 105**C21orf105**corticotropin releasing hormone**CRH**cyclin-dependent kinase inhibitor 1A**p21**disintegrin and metalloproteinase domain-containing protein 12**ADAM12**endogenous retrovirus group W, member 1**ERVWE1**endoglin**ENG**epidermal growth factor-like protein 7**EGFL7**fms-related tyrosine kinase 1**sFlt-1**glial cells missing**GCM1**glutathione peroxidase**GPx**growth hormone 2**GH2**heme oxygenase 2**HO-2**heme oxygenase-1**HO-1**hypoxia inducible factor 1, alpha subunit**hif1α**Inhibin A**Inhibin A**insulin-like growth factor 1**IGF1**Insulin-like growth factor 1 receptor**IGF1R**insulin-like growth factor 2**IGF2**Insulin-like growth factor binding protein 2**IGFBP2**KiSS-1 metastasis-suppressor**KiSS1**pappalysin 2**PLAC3**pentraxin 3*, *long**PTX3**placenta protein 13**PP13**placental growth factor**PlGF**placentallactogen**CSH1 hPL**placenta-specific 1**PLAC1**placenta-specific 4**PLAC4**plasminogen activator inhibitor type 1**PAI-1**plasminogen activator, tissue**t(PA)**pregnancy-associated plasma protein-A**PAPP-A**pregnancy-specific beta 1 glycoprotein**PSG1**salvador homolog 1protein**SAV1**selectin P**SELP**superoxide dismutase**SOD**tenascin XB**TNXB2**transforming growth factor, beta 1**tgfb1**trophoblast glycoprotein**TPBG**vascular endothelial growth factor A**VEGF*
